# Pathophysiological Studies of Monoaminergic Neurotransmission Systems in Valproic Acid-Induced Model of Autism Spectrum Disorder

**DOI:** 10.3390/biomedicines10030560

**Published:** 2022-02-27

**Authors:** Hsiao-Ying Kuo, Fu-Chin Liu

**Affiliations:** 1 Institute of Anatomy and Cell Biology, National Yang Ming Chiao Tung University, Taipei 11221, Taiwan; 2 Institute of Neuroscience, National Yang Ming Chiao Tung University, Taipei 11221, Taiwan

**Keywords:** valproic acid, autism spectrum disorder, serotonin, dopamine, norepinephrine, histamine, neurodevelopment

## Abstract

Autism spectrum disorder (ASD) is a neurodevelopmental disorder with complex etiology. The core syndromes of ASD are deficits in social communication and self-restricted interests and repetitive behaviors. Social communication relies on the proper integration of sensory and motor functions, which is tightly interwoven with the limbic function of reward, motivation, and emotion in the brain. Monoamine neurotransmitters, including serotonin, dopamine, and norepinephrine, are key players in the modulation of neuronal activity. Owing to their broad distribution, the monoamine neurotransmitter systems are well suited to modulate social communication by coordinating sensory, motor, and limbic systems in different brain regions. The complex and diverse functions of monoamine neurotransmission thus render themselves as primary targets of pathophysiological investigation of the etiology of ASD. Clinical studies have reported that children with maternal exposure to valproic acid (VPA) have an increased risk of developing ASD. Extensive animal studies have confirmed that maternal treatments of VPA include ASD-like phenotypes, including impaired social communication and repetitive behavior. Here, given that ASD is a neurodevelopmental disorder, we begin with an overview of the neural development of monoaminergic systems with their neurochemical properties in the brain. We then review and discuss the evidence of human clinical and animal model studies of ASD with a focus on the VPA-induced pathophysiology of monoamine neurotransmitter systems. We also review the potential interactions of microbiota and monoamine neurotransmitter systems in ASD pathophysiology. Widespread and complex changes in monoamine neurotransmitters are detected in the brains of human patients with ASD and validated in animal models. ASD animal models are not only essential to the characterization of pathogenic mechanisms, but also provide a preclinical platform for developing therapeutic approaches to ASD.

## 1. Introduction

Autism spectrum disorder (ASD) is a devasting neurodevelopmental disease with an increasing prevalence of ~18.5 per 1000 people [1]. The etiology of ASD is highly heterogeneous with genetic and environmental roots. The core syndromes of ASD patients are deficits in social communication/interaction and restrictive/repetitive behavior [2]. Because of its complicated and heterogeneous etiology, the pathological mechanisms are not yet fully characterized, which prevents the development of effective therapy for ASD.

### 1.1. Animal Models of ASD

ASD is a complex and heterogeneous neurodevelopmental disorder. Genetic mutations of genes involved in synaptic wiring and neurotransmission in the development and function of neural circuits have been linked to ASD pathogenesis [3,4]. Genetic animal models of ASD provide research platforms to investigate ASD pathophysiology caused by mutations of ASD-risk genes, including *MECP2, SHANK3, NLGN3*, etc. [3,4,5]. As developing brains are vulnerable to environmental insults, environmental factors also play a significant role in the pathogenesis of ASD. It has been estimated up to 40–50% of the variance in ASD pathogenesis is influenced by environmental factors [6]. Maternal exposure to pharmacological reagents, heavy metals, and vaccination against virus infection has been implicated in the etiology of ASD [6].

### 1.2. VPA-Induced ASD-Like Animal Models

Valproic acid (VPA) is prescribed medicine for treating epilepsy, migraine, and mood disorders [7]. However, clinical studies indicate that children with maternal exposure to VPA have a higher chance of developing ASD [8]. Subsequent animal studies confirm that maternally VPA-treated offspring develop prominent ASD-like phenotypes resembling the core syndromes of human ASD patients. 

Most VPA-induced animal models are established in the rodent owing to the easy accessibility to rodents in the laboratory setting. VPA-induced models have also been reported in non-human primates [9,10]. The time windows of VPA administration in producing offspring with ASD-like phenotypes are from neural tube closure to neurogenesis, i.e., embryonic (E) day 9-E12.5 in pregnant rats (Tables 1 and 2) [11,12,13]. Time windows of VPA exposure appear to critically influence the developmental consequences in the VPA models. Previous studies have compared the teratogenic effects of VPA in different time windows of exposure. For example, Rodier et al. have found that VPA exposure at E11.5, E12, or E12.5 results in differential vulnerabilities of cranial nerves in the brains of offspring [13]. Animals exposed to VPA at different gestational time windows also show distinct developmental trajectories and abnormalities in cortical and brainstem regions [14]. At the behavioral level, Kim et al. have reported that VPA exposure at E12, but not at E7, E9.5, and E15, causes significant ASD-like social abnormalities, suggesting that E12 is a critical period for inducing ASD-like phenotypes [15]. 

Intriguingly, postnatal VPA administration during the first two weeks after birth causes ASD-like behavioral phenotypes in rodents as well [16,17,18], suggesting that postnatal developmental processes, including synaptogenesis, myelination, and synaptic pruning are also vulnerable to VPA exposure. Therefore, ASD-like phenotypes could be induced by VPA administrated at either gestational or early postnatal stages. 

Regarding the issue of gender, the prevalence of human ASD patients among males is ~4.3 times higher than among females [1]. Most studies use male animals in VPA-induced ASD model studies. However, some groups also include females and found ASD phenotypes in female animals as well (Tables 1 and 2). To investigate sexual dimorphism of VPA effects, several studies have compared gender-specific alterations in maternally VPA-treated offspring [19,20,21,22,23]. Evidence shows gender-specific changes in the immune responses [19,20,22,24], cell density of cerebellum [23,25], network connectivity [26], anandamide signaling [27], attentional processing [28], and composition of gut microbiota [29] in maternally VPA-treated animal offspring. These sexual dimorphisms may be related to histone deacetylase (HDAC) inhibition by VPA, as Konopko et al. have reported differential epigenetic modification of *Bdnf* genes in maternally VPA-treated fetal brains of different genders [24]. In general, maternally VPA-treated male offspring have more severe ASD-like phenotypes compared to female offspring.

With respect to the dosage of VPA administration, maternal VPA exposure affects the nervous systems in a dose-dependent manner. Magnetic resonance imaging (MRI) studies have shown dose-dependent changes in networks of the primary motor cortex-premotor cortex and the primary motor cortex-supplementary motor area in humans, suggesting that VPA induces changes in specific neural circuits [30,31]. The higher dose of the expecting mother taking VPA, the higher risk of the offspring being diagnosed with ASD [32]. Frisch et al. have reported that offspring of rat dams receiving a high dose of VPA (720 mg/kg) daily during the entire pregnancy show the most severe phenotype of impaired learning and memory. In the same study, MRI data show that compared to a medium dose of VPA (470 mg/kg), the higher dose of maternal treatment of VPA (720 mg/kg) induced more significant reductions in cortical and brainstem regions of offspring [33]. Prenatal administration of a lower dose of VPA (200 mg/kg) did not induce ASD-like behavioral phenotypes [16]. Therefore, for the experimental induction of ASD-like behavioral phenotypes without severe developmental retardation in the offspring, VPA administrated at the dosage of 300–600 mg/kg in pregnant rodents and non-human primates are mostly used (Tables 1 and 2) [9,34,35,36,37]. 

### 1.3. Potential Mechanisms Underlying VPA-Induced ASD-Like Pathophysiology 

It is yet unclear how maternal VPA exposure induces ASD pathophysiology. VPA is known to have multiple pharmacological properties. Firstly, VPA is a simple branched-chain fatty acid (2-propylpentanoic acid) that can directly inhibit voltage-gated sodium channels and suppress the high-frequency firing activity of neurons. VPA also can indirectly inhibit GABA transferase resulting in increased GABA neurotransmission in the brain [38]. Note that the imbalanced excitatory/inhibitory (E/I) ratio in neural circuits has been proposed as a prevailing pathogenic mechanism of ASD [39,40]. Hence, VPA may reduce the excitability of neural circuits by inhibiting GABA uptake, leading to disrupted E/I balance, which may account for ASD phenotypes. In supporting this hypothesis, previous studies show that abnormal E/I balance and aberrant synaptic transmission have been found in the medial prefrontal cortex (mPFC), primary somatosensory cortex, amygdala, and dorsal raphe nucleus of maternally VPA-treated rodent offspring [41,42,43,44,45,46]. Furthermore, drugs that regulate neuronal excitability can partially rescue ASD-like phenotypes in VPA-treated animals [37,47], suggesting that E/I imbalance resulting from VPA treatment may be a key mechanism of ASD pathogenesis.

Secondly, VPA functions as a non-specific histone deacetylase (HDAC) inhibitor that can affect neural development by chromosome remodeling [6,7,8]. VPA-induced changes in neural development have been reported in many studies and discussed by several review articles [12,35,48]. The epigenetic effects of VPA have been confirmed by VPA-induced site-specific epigenetic alterations in rodents [49,50,51] and non-human primates [9], suggesting that VPA-mediated epigenetic modifications may contribute to VPA-induced ASD-like pathophysiology. Several genome-wide screening studies have identified the alteration of genetic networks by VPA. In non-human primates, prenatal VPA administration changes the expression of human ASD-associated genes and the gene expression patterns that are related to synaptogenesis, critical period, neural plasticity, and E/I balance, including *CADM1*, *LRRTM4*, *GRIN1,* and *GRIN2A* in marmoset infant brains [52]. Another RNA sequencing study has investigated transcriptome profiles in maternal VPA-treated marmoset embryos and found marked alterations in the gene expression pattern of neuronal development, including axon guidance and calcium signaling [9]. In rodent models, maternal VPA treatment induces changes in circadian rhythm- and extracellular matrix-related genes in the mPFC [53]. Altered gene expression profiles in neural development and function, cellular development and function, cell death, and immune system are also detected in the amygdala of maternally VPA-treated offspring brains [54]. Proteomics study has further found changes in protein expression profiles in synaptic function, energy metabolism, cytoskeleton and neuropsychiatric disorders in the cerebral cortex of maternally VPA-treated rat offspring [55]. Note that molecular and cellular changes in the VPA-induced animal models are similar, but not identical to those in idiopathic ASD patients.

On the other hand, VPA-induced changes in the immune system are of particular interest, because maternal immune activation during embryonic stages has been proposed as a pathogenic mechanism of ASD [56]. Inflammatory responses are detected in human ASD brains, including elevated levels of inflammatory biomarkers and activation of astrocytes and microglia [57]. Evidence suggests abnormalities in the interaction of the microbiota–gut–brain axis and the immune system in ASD brains [58], highlighting the multifaceted and potential pathogenic role of inflammation in ASD pathogenesis. In VPA-treated animals, marked signs of neuroinflammation have been detected, including elevated levels of reactive oxygen species, NFκB, and pro-inflammatory cytokines [59]. Moreover, anti-inflammatory drugs could alleviate ASD-like phenotypes in maternally VPA-treated animal offspring [37,47]. In light of these findings, we will discuss how monoamines interplay with the microbiota–gut–brain axis and the immune system later.

### 1.4. Advantages and Limitations of VPA-Induced ASD-Like Animal Models

Since the first rodent model in the 1970s, VPA exposure in experimental animals has been widely used as an ASD model to study the ASD etiology of environmental risk factors. The major advantage of studying VPA models is that the VPA-exposed animals develop robust ASD-like phenotypes resembling the core symptoms of ASD patients [7,11,12,35]. Its simple experimental protocols also allow it to be widely adopted as an animal model for studying ASD pathophysiology from molecular, cellular to behavioral levels for decades [12,35,48,60,61]. Moreover, as VPA is an HDAC inhibitor that can influence chromosome remodeling, VPA animal models may help understand the epigenetic basis of ASD etiology. 

The limitations of VPA models are, however, that VPA exposure is one of many environmental risk factors of ASD and maternally VPA-exposed ASD patients represent a small population of ASD. The VPA-induced pathophysiology may thus account for part of ASD pathophysiology. Nonetheless, because VPA-induced pathophysiology shares some similarities with ASD genetic mutation models, e.g., synaptopathy [4,62], the easy accessibility and the extensive knowledge built on this ASD model have been leveraged to screen for pharmacological reagents for treating ASD patients [37,47,63].

### 1.5. Dysfunction of Monoaminergic Neurotransmission in VPA-Induced ASD-Like Animal Models of ASD

**Figure 1:**
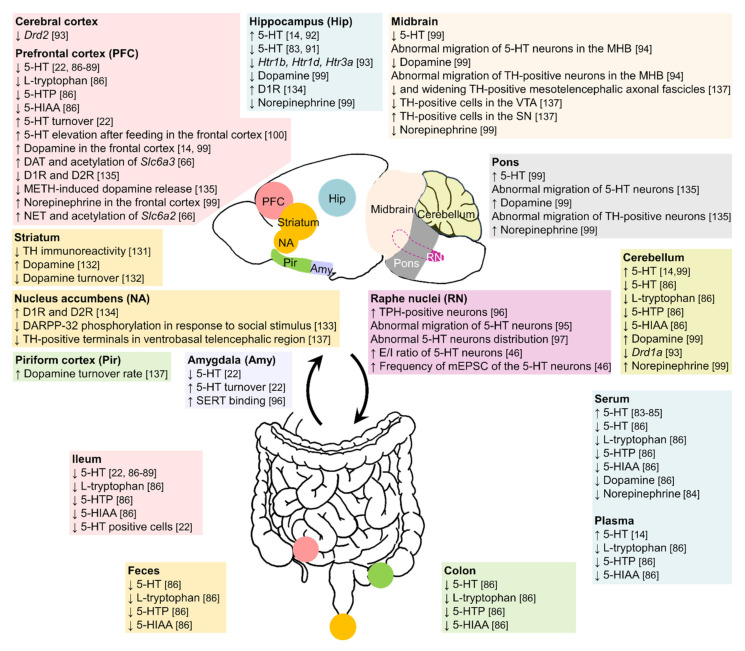
Alterations of monoaminergic systems in the brain and peripheral system of maternally VPA-treated rodent offspring. Schematic drawings illustrate pathophysiological changes in monoaminergic systems in the central nervous system and peripheral system of maternally VPA-treated rodent offspring. The bi-directional arrows indicate cross-talk between the central and peripheral systems. Abbreviations: ↑, increased; ↓, decreased; 5-HIAA, 5-hydroxyindoleacetic acid; 5-HT, 5-hydroxytryptamine (serotonin); 5-HTP, 5-hydroxytryptophan; D1R, dopamine D1 receptor; D2R, dopamine D2 receptor; DAT, dopamine transporter; DOPAC, 3,4-dihydroxyphenylacetic acid; E/I, excitatory/inhibitory; Htr, 5-hydroxytryptamine receptors; mEPSCs, miniature excitatory postsynaptic currents; METH, methamphetamine; MHB, midbrain-hindbrain boundary; NET, norepinephrine transporter; SERT, serotonin transporter; SN, substantia nigra; TH, tyrosine hydroxylase; TPH, tryptophan hydroxylase; VTA, ventral tegmental area.

**Figure 2:**
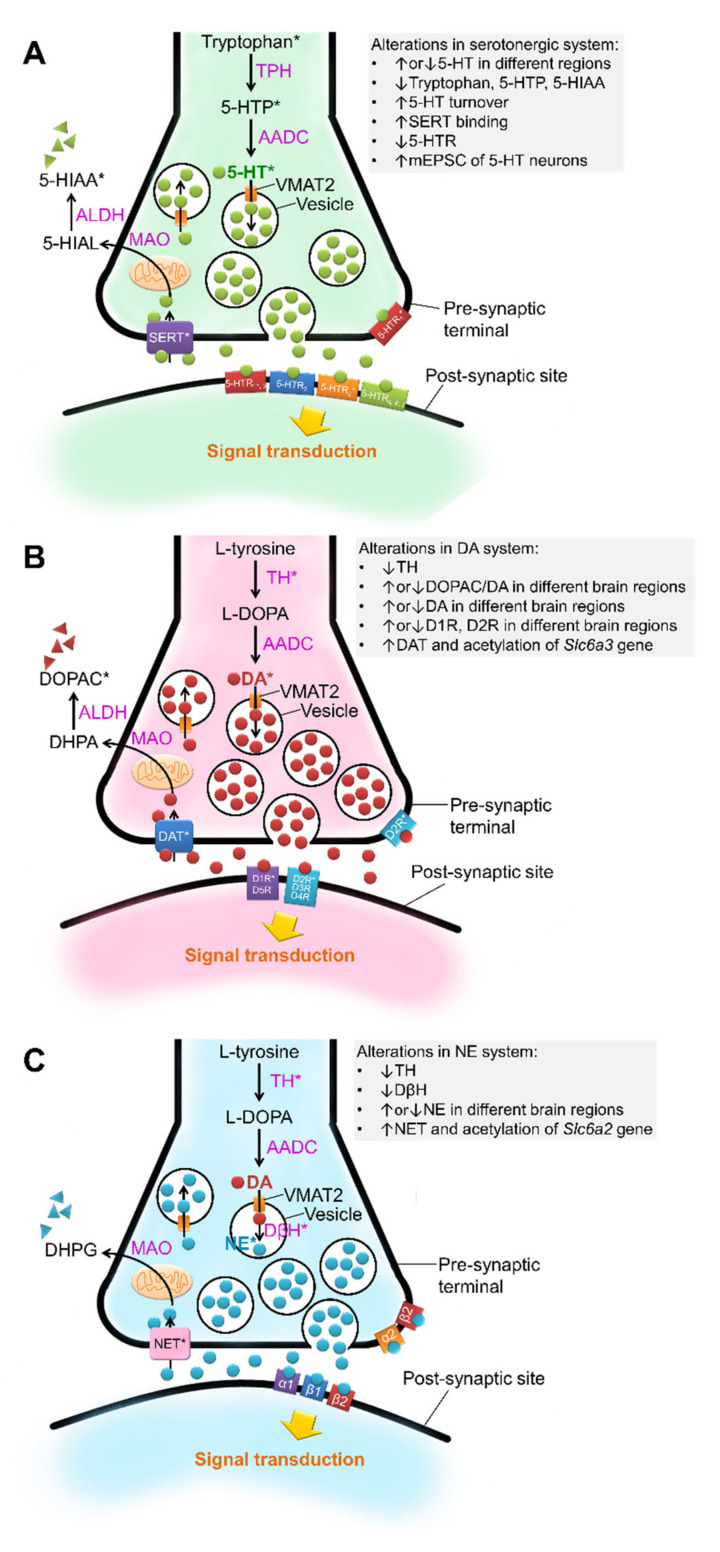
Schematic drawings illustrate monoaminergic neurotransmissions and their dysregulation in response to maternal VPA challenge. (**A**) Serotonergic neurotransmission. (**B**) Dopaminergic neurotransmission. (**C**) Norepinephrinergic neurotransmission. The neurotransmission molecules that are altered by maternal VPA treatments are indicated by asterisk*. Abbreviations: ↑, increased; ↓, decreased; 5-HIAA, 5-hydroxyindoleacetic acid; 5-HIAL: 5-hydroxyindole acetaldehyde; 5-HT, 5-hydroxytryptamine (serotonin); 5-HTP, 5-hydroxytryptophan; 5-HTR, 5-hydroxytryptamine receptor; AADC, aromatic L-amino acid decarboxylase; ALDH, aldehyde dehydrogenase; D1R, dopamine D1 receptor; D2R, dopamine D2 receptor; D3R, dopamine D3 receptor; D4R, dopamine D4 receptor; D5R, dopamine D5 receptor; DA, dopamine; DAT, dopamine transporter; DβH, dopamine-β-hydroxylase; DHPG, dihydroxyphenylglycol; DOPAC, 3,4-dihydroxyphenylacetic acid; MAO, monoamine oxidase; mEPSCs, miniature excitatory postsynaptic currents; METH, methamphetamine; NE, norepinephrine; NET, norepinephrine transporter; SERT, serotonin transporter; TH, tyrosine hydroxylase; TPH, tryptophan hydroxylase; VMAT, vesicular monoamine transporter.

Alterations of monoaminergic neurotransmitter systems have been detected in many brain regions as well as in the peripheral system in ASD pathophysiology (Figure 1; Tables 1 and 2). Unlike the classical neurotransmitters of glutamate and GABA that directly dictate neuronal activity, monoamines are neuromodulators that modulate the excitability of neurons by regulating synaptic neurotransmission (Figure 2; Tables 1 and 2).

Monoamine-modulating drugs are so far the available medications for treating ASD-associated syndromes. The atypical antipsychotics, risperidone and aripiprazole, are U.S. Food and Drug Administration (FDA)-approved clinical drugs for ASD. Risperidone and aripiprazole pharmacologically act on dopamine (DA) and serotonin of the monoaminergic systems. In supporting the importance of the monoaminergic systems for ASD, several studies have shown that antipsychotics targeting monoaminergic systems are effective in improving maternal VPA-induced ASD-like abnormalities [64,65,66,67].

The neural development of monoaminergic systems protracts a long time from embryonic to postnatal stages. The prolonged developmental time frame makes it vulnerable to pathological alterations that are induced by genetic and epigenetic challenges related to ASD. In the present review, we focus on the VPA-induced ASD model, because it has been widely adopted to investigate the neuropathophysiology of ASD and used as an experimental platform to develop therapeutic reagents [35,37,68]. We begin with an overview of the neural development and neurochemical property of each monoamine neurotransmitter, we then discuss the abnormalities of ASD in human patients and VPA-induced animal models. Schematic summary of the monoaminergic neurotransmission-related endophenotypes and other pathophysiological changes in the central nervous system and peripheral system of VPA-treated animals are illustrated in Figure 1 and Figure 2 and listed in Tables 1 and 2. Schematic drawings of synaptic transmission of monoaminergic neurotransmissions and VPA-induced dysfunction of monoaminergic neurotransmission are illustrated in Figure 2.

## 2. Alterations of Monoaminergic Systems in VPA-Induced ASD Models

### 2.1. Serotoninergic Systems

#### 2.1.1. Neural Development of Serotonergic Neurons

In the central nervous system, the majority of serotonergic neurons are localized in the raphe nuclei. All the serotonergic neurons are derived from the developing rhombencephalon. Serotonergic progenitors are generated in the isthmus and p3 domain of the ventral hindbrain. Serotonergic neurons undergo neurogenesis at E 9.5–E12 in mice and E10.5–E13 in rats. Shortly after cell mitosis, differentiating serotonergic neurons begin their migration as early as E10 [69]. The early-born neurons migrate to form the anterior cluster of serotonergic neurons (B5–B9) that extensively innervate the forebrain, midbrain, cerebellum, and raphe nuclei themselves. The late-born neurons migrate caudally to form the posterior cluster of serotonergic neurons (B1–B4) that innervate the brainstem and spinal cord [70]. Upon the end of differentiation, serotonergic neurons produce 5-hydroxytryptamine (5-HT, i.e., serotonin) not only for neurotransmission, but also for facilitating the maturation of themselves. Along with axonal outgrowth and navigation, serotonergic neurons start to release 5-HT during axonal outgrowth as early as E16.5. The extensive and prolonged axonal innervations of serotonergic neurons are not completed until several weeks after birth [71].

#### 2.1.2. Neurochemical Properties of Serotonin

Tryptophan is the precursor for the synthesis of serotonin. Tryptophan hydroxylase (TPH), the rate-limiting enzyme found only in serotonergic neurons, converts tryptophan into 5-hydroxytryptophan (5-HTP). Then, aromatic L-amino acid decarboxylase (AADC) converts 5-HTP into 5-HT. Cytoplasmic 5-HT is packed into synaptic vesicles through vesicular monoamine transporter 2 (VMAT2), where it is stored until being released into the synaptic cleft by exocytosis. The neurotransmission activity of 5-HT in the synaptic cleft is terminated by the serotonin transporter (SERT) that reuptakes 5-HT back to presynaptic terminals [70]. Because SERT is critical to 5-HT activity, the kinetics of 5-HT neurotransmission is governed by the level of SERT. 5-HT is metabolically degraded by monoamine oxidase (MAO) and converted into 5-hydrozy-indoleacetaldehyde, which is subsequently oxidized by aldehyde dehydrogenase to derive 5-hydroxyindoleacetic acid (5-HIAA). See Figure 2 for the summary of serotoninergic neurotransmission.

#### 2.1.3. Clinical Evidence of Abnormalities of Serotoninergic Systems Related to ASD

Clinical evidence of dysregulated serotoninergic systems in ASD patients is yet disputed. The first clinical study has reported hyperserotonemia in early-onset ASD patients [72]. Consistent with this report, a meta-analysis study has revealed elevated levels of 5-HT in the blood of 25.4% of ASD patients [73]. Another group has also reported an increased number of dystrophic 5-HT axons in different regions of human ASD brains [74,75]. Despite the evidence of hyperserotonemia as reported in some clinical studies, postmortem studies indicate different aspects of serotonin dysfunction in ASD pathophysiology. Significant reductions in receptor-binding density of two 5-HT receptors, G_i_-coupled 5-HT_1A_ and G_q_-coupled 5-HT_2_, were found in the posterior cingulate cortex and fusiform gyrus of ASD patients [76]. Moreover, abnormally increased and decreased levels of serotonin receptors were, respectively, observed in ASD patients with and without seizure histories [76]. Age-dependent alterations of the serotonergic systems have recently been identified, especially in the anterior cingulate cortex [77]. In addition to neuroanatomical alterations, genetic linkage studies have observed that SERT variants are correlated with platelet hyperserotonemia and impaired social communication in ASD patients [78,79,80]. Animal studies have found abnormal serotonergic neurotransmission in ASD-like pathophysiology as well. Normal serotonin levels are critical to the maintenance of E/I balance in cortical neurons, and restoration of serotonin levels can alleviate ASD-like phenotypes [81]. The causal relationship between the ASD-associated SLC6A4/SERT Ala56 coding variant and hyperserotonemia has been demonstrated in SLC6A4/SERT Ala56 gain-of-function transgenic mice that exhibited ASD-like behaviors [82].

#### 2.1.4. Abnormalities of Serotonergic Systems in VPA-Induced ASD Models

In the peripheral system, previous studies have found elevated 5-HT levels in serum, plasma or gastrointestinal tract of VPA-treated rodents, which support the general hyperserotonemia theory of ASD etiology [14,83,84,85]. However, other studies have reported the reduction in 5-HT-positive cells in the ileum, 5-HT levels and its precursors (tryptophan and 5-HTP), and metabolites (5-HIAA) in both the peripheral system and brain [22,86,87,88,89]. Note that short-term depletion of 5-HT precursor tryptophan deteriorates repetitive behavior and elevates anxiety status in ASD patients [90]. Given the complexity of clinical case studies, e.g., variations in the genetic, comorbidity, medication, and diet, it remains to be clarified how dysfunction of peripheral 5-HT takes part in ASD pathophysiology.

In the central nervous system, alterations of 5-HT levels have been detected in several brain regions of VPA-treated offspring. Serotonergic neurons of the median raphe nucleus primarily project to the hippocampus, whereas serotonergic neurons of the dorsal raphe nuclei preferentially project to the prefrontal cortex, basal ganglia, and amygdala [70]. In contrast to the more consistent results of VPA-induced 5-HT levels in the regions that are innervated by the dorsal raphe nucleus, the VPA-induced alterations of 5-HT levels are varied in the hippocampus [14,18,83,91,92]. The dorsal and median raphe nuclei may differ in vulnerabilities to VPA treatments. Reduced serotonin receptors have also been found in the hippocampus [93]. In other brain regions, alterations in 5-HT levels are more consistent. In VPA-treated rodents, increased 5-HT levels are detected in the cerebellum and pons; however, reduced 5-HT levels are detected in the prefrontal cortex, midbrain, and amygdala. Considering developmental changes in VPA-treated rodents, administration of VPA during E9-E12.5 is at the time window of neurogenesis, differentiation, and migration of serotonergic neurons in the raphe nuclei. Kuwagata et al. have reported differential vulnerabilities of 5-HT-positive neurons to different time windows of maternal VPA administration. They found abnormal migration of 5-HT-positive neurons in rats receiving VPA at E11, but not in rats receiving VPA at E9 [94]. Wang et al. found an increased number of 5-HT-positive neurons in the raphe magnus nucleus (caudal part of raphe nuclei), but Miyazaki et al. report no significant changes in the total number of 5-HT-positive cells in the raphe nuclei [95,96]. These two studies are different at VPA dose and the timing of VPA injections. The discrepancy of these findings may be partly explained by VPA-induced abnormal migration in the raphe nuclei as reported by other groups [94,95,97]. Abnormal differentiation of serotonergic neurons has been reported in the embryos of VPA-treated zebrafish [98]. Because VPA is an HDAC inhibitor that can regulate chromatin remodeling, it would be of interest to study how VPA-induced epigenetic changes in chromatin remodeling affect the development of serotonergic neurons.

#### 2.1.5. Behavioral Phenotypes Related to Abnormal Serotonergic Systems in VPA-Induced ASD Models

At the functional level, Wang et al. (2018) reported abnormal excitatory/inhibitory balance and mEPSCs of 5-HT neurons in the dorsal raphe nucleus of VPA-treated brains, implicating a role of 5-HT in the modulation of excitatory/inhibitory balance of neuronal activity [46]. With respect to behavioral phenotypes, most of the studies have found abnormal social behaviors along with abnormalities in serotonergic systems [18,22,83,84,86,87,88,89,91,92,96,99]. Impaired social communication is a hallmark of ASD symptoms, and defective social interaction also occurs in VPA-induced ASD animals. Other ASD-like behavioral phenotypes in serotonergic deficient VPA mice are increased repetitive behaviors and anxiety levels, abnormal sensation, delayed developmental milestones, and impaired memory [18,83,84,86,87,88,89,92,96,99]. By contrast, the findings of locomotion are inconsistent in that some studies have reported reduced locomotion [18,84,86], but other studies found elevated locomotion [87,88,89,91,92,100]. This inconsistency cannot be explained by species of animals or dosage of VPA. An interesting finding is that abnormal circadian rhythm and activity were found in rats that maternally received VPA at E9.5. Note that a high proportion of patients with autism show sleep disturbance, especially insomnia [101,102]. Regarding the causality of abnormal serotonergic systems and behavioral abnormalities, Wang et al. (2013) have treated VPA-induced ASD rats with 5-HT_1A_ receptor agonists during behavioral tests. They found that the treatment of 5-HT_1A_ receptor agonists could restore social impairments and improve extinction of fear memory, suggesting that abnormal serotonergic signaling may contribute to abnormal social behaviors and memory deficits in VPA-treated rodents [96]. In the future, more studies are required to further clarify the causal relationship between behavioral phenotypes and serotonergic abnormalities (Table 1).

#### 2.1.6. Microbiota–Gut–Brain Axis and Serotoninergic Systems in VPA-Induced ASD Models

Peripheral 5-HT is mainly produced by enterochromaffin cells from dietary tryptophan in the gastrointestinal tract, and is taken up by platelets or SERT on serotoninergic presynaptic terminals. Peripheral 5-HT regulates gastrointestinal motility and is involved in immune function [103]. An intriguing issue is how peripheral 5-HT affects the central nervous system, given that 5-HT in the central and peripheral nervous systems is separated by the blood–brain barrier. 5-HT may influence enteric nerves, vagal afferent activity, or inflammatory responses, and then modulate the central nervous system indirectly [58]. Another potential mechanism of peripheral 5-HT influence neurodevelopmental process is mediated by the microbiota–gut–brain axis. Previous studies have demonstrated the causal relationship of the microbiota–gut–brain axis and the pathogenesis of ASD, and ~70% of ASD patients show comorbid gastrointestinal disturbances [58]. 5-HT has been shown to modulate bacterial motility and gene expression profile of bacteria in vivo [58,104], indicating complex feedback interactions between 5-HT and microbiota in the gastrointestinal tract. Conversely, the microbiota is known not only to synthesize 5-HT, but also to modulate the serotonergic system in the gastrointestinal tract of the host, suggesting that 5-HT may be a communication molecule of gut–brain interactions. In germ-free mice, TPH (rate-limiting enzyme of 5-HT) is upregulated, and the 5-HT level in the hippocampus is reduced with enhanced anxiety [105,106]. Animal studies have found the abnormal composition of microbiota in ASD-like models, including VPA-treated rodents [84,85,86]. The VPA-treated rodents also showed altered 5-HT, L-tryptophan, 5-HTP, and 5-HIAA levels in serum, colon, feces, cerebellum, and prefrontal cortex [84,85,86]. Given the important role of the microbiota–gut–brain axis in ASD pathophysiology, it would be of interest to see if the modulation of the gut–brain axis by serotonin may be involved in the etiology of ASD.

### 2.2. Catecholamines

#### 2.2.1. Neural Development of Catecholaminergic Neurons

DA and norepinephrine (NE) are the primary catecholamines that are released from groups of catecholamine neurons in different brain regions (A1–A17). An additional three adrenaline-containing groups (C1–C3) are distributed in the telencephalon, mesencephalon, and rhombencephalon [107]. Catecholamines are important neurotransmitters that are essential to brain function. Dysfunction of catecholamines is involved in the pathophysiology of neurodevelopmental and neurodegenerative disorders, including ASD and Parkinson’s disease.

Although dopaminergic neurons comprise a small population of neurons in the brain, they profoundly control brain function through the extensive innervations of their network [107]. Among the different groups of dopaminergic neurons, substantia nigra pars compacta (SNc, A9) and ventral tegmental area (VTA, A10) play important roles in the control of motor, reward, motivation, and emotion. Progenitors of dopaminergic neurons are originated from the ventral midline of the floor/basal plate that is located near to the midbrain–hindbrain boundary (MHB), or isthmus. In mice, SNc dopaminergic neurons are generated before E11 and their neurogenesis is peaked at E11–E12, whereas the peak of neurogenesis of VTA dopaminergic neurons occurs at E12–E13 [108]. After exiting the cell cycle progression, midbrain dopaminergic neurons migrate radially and/or tangentially from the ventricular zone toward the pial surface, and this migratory process continues until the first week after birth. [109,110].

On the other hand, cell bodies of norepinephrinergic neurons are clustered in the medulla oblongata, pons, and midbrain. The locus coeruleus (LC) is known as the major source of NE in the brain, which is located near the floor of the fourth ventricle [70]. Progenitors of LC neurons are mainly derived from rhombomere 1, and LC neurons are born early and migrate toward the basal plate before E10.5 [111]. Neurogenesis of norepinephrinergic neurons occurs predominantly between E10.5–E12.5. Norepinephrinergic neurons are functionally active by birth [112], and they gradually mature during postnatal periods [113].

#### 2.2.2. Neurochemical Properties of Catecholamines

All the catecholamines are derived from L-tyrosine. The rate-limiting enzyme, tyrosine hydroxylase (TH), converts L-tyrosine to L-DOPA, and AADC subsequently converts L-DOPA to DA. For neurons that synthesize NE or epinephrine, dopamine-β-hydroxylase catabolizes DA into NE, and phenylethanolamine N-methyltransferase further converts NE into epinephrine. The degrative metabolism of catecholamines is mediated by MAO and catechol-O-methyltransferase (COMT). DA and NE are transported into synaptic vesicles by VMAT2. After being released into the synaptic cleft, NE and DA are recycled back into the presynaptic terminal, respectively, through the uptake by norepinephrine transporter (NET) and dopamine transporter (DAT). The DA receptor family consists of two members, D_1_-like and D_2_-like receptors. Activation of D_1_-like receptor increases cAMP levels, whereas activation of D2-like receptor decreases cAMP levels in DA responsive cells. As for the NE receptor, NE binds to three NE receptor families (α1, α2, β) [70]. See Figure 2 for the summary of catecholaminergic neurotransmission.

#### 2.2.3. Clinical Evidence of Abnormalities of Catecholaminergic Systems Related to ASD

An early study documented dysregulation of catecholamines in ASD patients whose plasma level of NE is increased, but dopamine-β-hydroxylase is decreased [114]. Clinical study has shown reduced DA level in the mPFC of medication-free ASD patients, which suggests an aberrant function of the dopaminergic systems in ASD [115]. Genetic linkage studies have revealed that mutations of many dopaminergic systems regulators are associated with ASD, including dopamine receptor *DRD1* [116], *DRD2* [117], *DRD3* [118], *DRD4* [119], and *DAT* [120,121]. The dopaminergic systems are of particular interest with respect to ASD pathophysiology, because it participates in the reward system that is involved in social motivation and social interaction [122,123]. Because of the multifaceted roles of the dopaminergic systems in reward prediction, decision making, and motivation [124,125,126] that are affected in ASD patients, it is imperative to study the pathophysiological changes in dopaminergic systems in ASD.

As for the norepinephrinergic systems, previous studies have reported abnormalities of resting-state functional connectivity in LC and elevated tone of NE activity in ASD children [127,128]. Another positron emission tomography study found a correlation between the binding signals of D1R and NET and ASD symptoms [129]. Moreover, antipsychotic drugs targeting NE receptors have been shown to have beneficial effects on ASD symptoms [130].

#### 2.2.4. Abnormalities of Catecholaminergic Systems in VPA-Induced ASD Models

Alterations of dopaminergic systems have been extensively reported in studies of VPA-induced ASD animal models. Reduction in the rate-limiting enzyme of TH has been found in the striatum of VPA-treated rats [131]. The reduced TH level presumably would result in decreased DA level in the striatum. However, another study has reported an elevated DA level in the striatum of VPA-treated mice [132]. In fact, the increased DA levels were found not only in the striatum, but also in the frontal cortex, cerebellum, and pons [14,99,132]. By contrast, decreased DA levels were found in the hippocampus, midbrain, and serum of VPA-treated animals [86,99]. The variations in the changes in DA levels in different brain regions may result from the differences in DA turnover and/or the expression of DA-related signaling molecules in different brain regions, including DA receptor, acetylation of DAT, and phosphorylation of DARPP-32 in response to social stimulus [66,93,133,134,135]. Along this line, an interesting question is whether the dopaminergic systems may be affected by the external environment. Indeed, elevated DA levels and reduced DA turnover were found in VPA mice raised in different social environments [132].

In the norepinephrinergic systems, reduced NE levels are detected in the hippocampus, midbrain, and serum; whereas increased NE levels are found in the frontal cortex, cerebellum, and pons [84,99]. Furthermore, increased NET expression and acetylation levels have been reported in VPA-treated rats [66]. Regarding VPA-induced changes in neuronal development, abnormal migration of TH-positive neurons have been found in the pons, and the boundary of the midbrain and hindbrain [94], which implicates that pathological changes in the catecholamine systems may result from VPA-induced developmental abnormalities.

#### 2.2.5. Neuroanatomical Phenotypes Related to Altered Catecholamine Systems in VPA-Induced ASD Models

In addition to altered metabolic, abnormal migration, and distribution of TH-positive DA neurons in the SNc and VTA have been found in VPA-treated rats and chickens [94,136]. Regarding dopaminergic projections, a recent study has used the tissue clearing iDISCO method combined with a laser light-sheet confocal microscope to investigate whole-brain dopaminergic axonal projections. Ádám et. al. (2020) have found reduced ventrobasal telencephalic projections from the VTA along with a reduced number of DA neurons in the VTA of VPA-treated mice. By contrast, an increased number of DA neurons in the substantia nigra was observed in VPA-treated mice. These findings suggest region- and cell-type specificity in VPA-induced pathophysiology and further suggest potential compensatory mechanisms among different dopaminergic neuronal populations in the midbrain [137].

#### 2.2.6. Behavioral Phenotypes Related to Altered Catecholamine Systems in VPA-Induced ASD Models

At the behavioral level, consistent behavioral phenotypes have been found along with catecholaminergic abnormalities in VPA-treated rodents, including reduced social behaviors, increased repetitive, depressive and anxious behaviors, and impaired memory [14,66,84,86,93,99,131,133,134,138]. Impaired cognitive flexibility and temporal processing have also been observed in VPA-treated rodents, implicating abnormal functions of the prefrontal cortex [131,132]. Reduced levels of hyperlocomotion in response to methamphetamine further support the abnormalities of dopaminergic systems in VPA-treated mice [135]. Risperidone and aripirazole are two atypical antipsychotics for alleviating ASD syndromes in VPA-treated mice, including impairments in social behaviors, vocalization, and recognition memory [64,65]. Note that risperidone inhibits D2R and 5-HT_2A_ receptors, whereas aripirazole acts as an agonist of 5-HT_2A_ receptor and also as a partial agonist of D2R. The causality between dopaminergic and behavioral phenotypes awaits further investigation. On the other hand, hyperactivity, repetitive behaviors, impaired social interaction, and recognition memory can be alleviated by atomoxetine, a blocker of NET. The neurochemical mechanisms by which altered catecholaminergic systems contribute to ASD pathogenesis require further studies.

#### 2.2.7. Altered Reciprocal Interactions of Microbiota and Catecholamine Systems in VPA-Induced ASD Models

Decreased levels of DA and NE in serum have been found in VPA-treated rats along with the abnormal composition of gut microbiota [84,86]. NE has been shown to regulate various aspects of bacteria, from bacterial growth, migration to the gene expression profile of bacteria [58]. Some bacteria are found to express monoamine transporter on their plasma membrane [139]. Other bacteria can convert host-derived inactive forms of NE and DA into biologically active forms [140], suggesting a role of microbiota in modulating the activity of catecholaminergic systems. The mechanisms underlying reciprocal interactions between peripheral catecholaminergic systems and microbiota–gut–brain axis and how dysfunctional microbiota–gut–brain axis affect central catecholaminergic systems await future studies.

### 2.3. Histamine

#### 2.3.1. Neural Development of Histaminergic Neurons

In the postnatal stages, histaminergic neurons are mainly distributed in the tuberomammillary nucleus of the hypothalamus, and they extensively innervate a variety of brain regions, including the cerebral cortex, hippocampus, preoptic area, striatum, and thalamus [141]. Histidine decarboxylase (HDC) is an enzyme that catalyzes the decarboxylation of histidine to form histamine. Neurogenesis of HDC-positive neurons in the tuberomammillary nucleus begins from E13-18 and is peaked at E16 in the rat brain [142]. Notably, there is a transient histaminergic system during prenatal brain development. Transient histamine-immunoreactive neurons are located in the mesencephalon, metencephalon, and rhombencephalon, and they are no longer detected at the end of embryonic stages in the rat brain. The peak of neurogenesis in the brain and the highest level of histamine coincide at ~E14, implicating a potential role of histamine in regulating neurogenesis in the rat brain [143]. Later studies have revealed a regulatory role of histamine in promoting neural proliferation, cell-type specific differentiation, and axogenesis [143]. It has also been reported that histamine systems can cross-talk with other neurotransmitter systems by forming heteroreceptors (see below) and by other unknown mechanisms. For example, the expression levels of dopamine, D2R, and D3R are increased in *HDC* knockout mice [144,145].

#### 2.3.2. Neurochemical Properties of Histamine

The histaminergic systems play important roles in modulating the sleep–wake cycle, reward, neuroinflammation, emotion, and learning and memory [146]. Histamine is converted from L-histidine by histidine decarboxylase (HDC), and is packaged into synaptic vesicles by VMAT2 before releasing. Although there are no high-affinity reuptake mechanisms for uptaking histamine, histamine N-methyltransferase (HNMT) is responsible for the clearance of histamine in the synaptic cleft [70,141]. In the brain, neuronal expression of histamine receptor H1 (H1R), histamine receptor H2 (H2R), and histamine receptor H3 (H3R) are engaged in the regulation of different neurological functions. For example, H1R and H3R signalings are important for regulating the sleep–wake cycle, whereas H2R signaling is involved in aggression [147].

#### 2.3.3. Clinical Evidence of Abnormalities of Histaminergic Systems Related to ASD

Clinical studies have linked abnormal histaminergic systems to ASD pathogenesis. Human postmortem brain study has found increased HNMT levels and histamine receptors in the dorsolateral prefrontal cortex of ASD patients [148], suggestive of altered histaminergic systems in ASD pathophysiology. The histaminergic system transiently but profoundly affects the developmental processes of other neural systems, including serotonergic and dopaminergic systems [144,149]. Furthermore, histamine not only modulates the proliferation and differentiation of neural stem cells, but also regulates the biological functions of astrocytes and microglia that are involved in neuroinflammation [144,149]. Taken together, histaminergic dysfunction may contribute to autistic abnormalities either directly as a neuromodulator or indirectly through affecting neuronal development (Table 2).

#### 2.3.4. Abnormalities in the Histaminergic System in VPA-Induced ASD Models

Similar to what is observed in clinical studies, abnormalities in the histaminergic system have been found in VPA-induced ASD-like animal models as well. Reduced levels of histamine receptor H3 (H3R), HDC and histaminergic neurons have been found in VPA-treated zebrafish [150]. Notably, H3R antagonists have therapeutic effects on VPA-treated mice in reducing inflammation and ASD-like behaviors [151,152,153]. H3R antagonists also alleviate ASD-like symptoms in other ASD animal models [154,155]. Given that Gi-coupled H3R can indirectly modulate other neurotransmitter systems by forming heteroreceptors, the involvement of histaminergic systems in ASD pathophysiology may have been underestimated. Until now, there is no study revealing alterations of histaminergic systems except VPA-treated zebrafish. No information is yet available for potential changes in the central and peripheral systems in mammals.

Histamine is not only synthesized in the central nervous system, but also in the peripheral mast cells and gastric enterochromaffin-like cells that play important roles in the microbiota–gut–brain axis [58]. Moreover, some microbiota in the gastrointestinal tract of the host also synthesize histamine. Although histamine hardly penetrates the blood–brain barrier, in addition to the microbiota–gut–brain axis, histamine may indirectly affect the nervous system via the immune system and the vagal system [58,156]. It is worthwhile to note that histamine is a key player in many immune responses, because maternal immune activation is a risk factor for offspring to develop ASD [56]. It is imperative to decipher the mechanisms by which histamine by itself and/or through regulating other neurotransmission systems contribute to the pathophysiology of ASD.

## 3. Conclusions

Over the decades of studies, the VPA-induced ASD-like animal models have provided important information about the pathophysiology of ASD. The monoaminergic systems, key players of neuromodulation in the brain, are extensively affected in the pathophysiology of ASD as demonstrated in the VPA-induced ASD model. In this review, we summarize and discuss the pathological changes in monoaminergic systems related to ASD etiology. Given the pharmacological nature of VPA that can regulate cellular signaling as well as chromatin remodeling as an HDAC inhibitor, the information gained from VPA model studies with respect to monoaminergic systems would not only help clarify the pathogenic trajectories of ASD at the cellular and epigenetic levels, but it may also help develop therapeutic reagents for ASD [37]. 

## Figures and Tables

**Table 1:** Summary of valproic acid (VPA)-induced teratogenicity in serotonergic systems.

Monoamines-Related Endophenotypes (Age of Animals during Analysis)	Other Molecular, Cellular and Physiological Phenotypes (Age of Animals during Analysis)	Behavioral Phenotypes (Age of Animals during Analysis)	Animals/ Gender	VPA Exposure	References
**Serotonergic System**					
↓ 5-HT in the hippocampus ↑ 5-HT in serum (> 3 m/o)	↑ Inflammation in colonic tissues ↓ Tight junction protein (claudin1 and occluding) in the colonic tissues (> 3 m/o)	↓ Social play behavior Abnormal olfactory habituation/dishabituation (11 wks–3 m/o)	Wistar rats/male	600 mg/kg, E12.5, i.p.	[83]
↑ 5-HT in serum (P28)	↑ Proinflammatory cytokines: IL-17A, TNF-α, IL-6 ↓ GABA in serum Abnormal gut microbiota	Delayed developmental milestones (P2-21) ↓ Locomotion ↓ Social behaviors ↑ Repetitive behavior (P28)	Wistar rats/male	600 mg/kg, E12.5, i.p.	[84]
↑ 5-HT in serum (N/A)	Abnormal gut microbiota Altered several metabolic pathways caused by microbial dysbiosis (P21, 28, 35)	↑ Anxiety ↓ Social behaviors (6 wks)	C57BL/6 mice/male	500 mg/kg, E12.5, i.p.	[85]
↑ 5-HT in the hippocampus, cerebellum, and plasma (P50)			SD rats/male	800 mg/kg, E9, oral feeding	[14]
↓ 5-HT and its precursors (L-tryptophan and 5-HTP) and final metabolites (5-HIAA) in the colon, feces, serum, cerebellum, and PFC (8 wks)	↓ GABA in serum, cerebellum, and PFC ↑ Glutamate in serum, cerebellum, and PFC ↓ Acetylcholine in serum and PFC Abnormal gut microbiota (8 wks)	↓ Novel object recognition ↓ Social behavior ↓ Locomotion ↑ Depression ↑ Repetitive behavior (8 wks)	Wistar rats/male	500 mg/kg, E12.5, i.p.	[86]
↓ 5-HT in the PFC and amygdala ↑ 5-HT turnover (5-HIAA/5-HT) in the PFC and amygdala ↓ 5-HT levels and numbers of 5-HT positive cells in the ileum (P28)	↑ Epithelial loss and intestinal inflammation in the ileum ↑ Neuroinflammation in the dorsal hippocampus (P28)	↓ Social interaction (P28)	BALB/c mice/both gender	500 or 600 mg/kg, E11.5, s.c.	[22]
↓ 5-HT in the PFC and ileum (P50)	↓ Gastrointestinal tract motility ↓ Activity of mitochondrial enzyme complex-I, II, V in PFC ↑ BBB permeability ↑ Oxidative stress ↑ Nitrosative stress (nitrate/nitrite) ↑ Inflammation in the brain and ileum ↑ Calcium (P50)	↓ Social behaviors (P44, 45) ↓ Exploratory behaviors (P47) ↑ Locomotion (P49) ↑ Repetitive behaviors (P46) ↑ Anxiety (P47, 48)	Wistar rats/male	500 mg/kg, E12.5, s.c.	[87,88,89]
↑ 5-HT in the hippocampus (P110)	↑ Inflammation: ↓Glutathione and catalase; ↑Total nitrite ↓ Cerebellar Purkinje cells ↑ Neurodegenerative chromatolysis in the cerebellum (P110)	Developmental delay (P9–12) Impaired nociceptive sensation ↑ Locomotion ↓ Rearing and hole poking ↑ Anxiety ↓ Social behaviors (P90–110)	Rats/male	600 mg/kg, E12.5, i.p.	[92]
↑ 5-HT in the hippocampus (P40)	↑ Oxidative stress ↓ Number of Purkinje cell layer in the cerebellum (P40)	↓ Locomotion ↓ Motor coordination ↓ Social behavior ↓ Spatial learning and memory ↑ Anxiety Impaired nociceptive sensation Delayed negative geotaxis (P40)	BALB/c mice/both gender	400 mg/kg, P14, s.c.	[18]
↓ 5-HT in the midbrain ↑ 5-HT in the cerebellum and pons (P22)	↑ Free amino acid in the frontal cortex (P22)	↓ Social behaviors ↓ Spatial memory (P21)	Albino rats/male	800 mg/kg, E12.5, oral feeding	[99]
↓ 5-HT in the hippocampus n.s. SERT and 5-HIAA among a variety of brain regions (P50)		↑ Locomotion ↓ Social behaviors (P31)	Wistar rats/male	500 mg/kg, E9, i.p.	[91]
↑ TPH-positive neurons in the raphe magnus nucleus (P28) ↑ SERT binding in the amygdala (P60)	↑ Amplitude of mEPSC in the lateral amygdala ↓ Paired pulse facilitation ratio in the thalamo-amygdaloid synapses (P35)	↓ Social behaviors Impaired fear memory extinction (P28–35)	SD rats/male	500 mg/kg, E12.5, i.p.	[96]
↑ Elevation of 5-HT after feeding in the frontal cortex (P56–105)		↑ Locomotion (P18) ↓ Body weight (4 wks) Abnormal circadian rhythm and activity (P28–43)	Wistar rats/both gender	800 mg/kg, E9.5. oral feeding	[100]
↓ Serotonin receptor, *Htr1b*, *Htr1d*, *Htr3a* mRNA in the hippocampus (P35)	Altered expression in genes encoding cholinergic and adrenergic receptors in the cortex and cerebellum (P35)	N/A	Wistar rats/both gender	800 mg/kg, E11, oral feeding	[93]
↓ or absent 5-HT neuronal differentiation in embryos (48 and 72 hpf)	Lack of Mauthner neurons ↓ Proneural gene *ascl1b* (28 and 32 hpf)	N/A	Zebrafish/N/A	0.625 mM, from 50% epiboly to 27 hpf, incubated in normal medium 0.625 mM, from 24 to 48 hpf, incubated in normal medium	[98]
Abnormal migration of 5-HT in adult dorsal raphe nucleus n.s. in number of 5-HT positive neurons (P50)	↓ Shh mRNA level at E9	N/A	Wistar rats/male	800 mg/kg, E9, oral feeding	[95]
Abnormal migration of 5-HT-positive neurons in the pons Abnormal navigation of 5-HT-positive neurons in the boundary of midbrain and hindbrain (E16)	Disorganization of cortical lamination (E16)	N/A	SD rats/both gender	800 mg/kg, E9 or E11, oral feeding	[94]
Abnormal 5-HT neurons distribution (dorsal tangential migration) in the rostral raphe nucleus (E15.5)	↓ *Shh* mRNA expression around the isthmus (E11.5)	N/A	Wistar rats/both gender	800 mg/kg, E9.5, oral feeding	[97]
↑ The excitation/inhibition ratio by enhancing glutamatergic synaptic transmission of the 5-HT neurons in the dorsal raphe nucleus ↑ Frequency of mEPSCs of the 5-HT neurons in the dorsal raphe nucleus (6–8 wks)	↓ Spike-timing-dependent long-term potentiation in the dorsal raphe nucleus (6–8 wks)	↑ Anxiety (8 wks) ↓ Body weight (P1)	Long Evans/male	400 mg/kg, E12.5, s.c.	[46]

All the animals in the studies listed in this table received a single administration of VPA. Abbreviations: ↑, increased; ↓, decreased; 5-HIAA, 5-hydroxyindoleacetic acid; 5-HT, 5-hydroxytryptamine (serotonin); 5-HTP, 5-hydroxytryptophan; BBB, blood–brain barrier; dpf, days post-fertilization; E, embryonic day; GABA, γ-Aminobutyric acid; hpf, hours post-fertilization; Htr, 5-hydroxytryptamine receptors; IL, interleukin; m/o, months old; i.p., intraperitoneal injection; mEPSCs, miniature excitatory postsynaptic currents; N/A, not applicable; P, postnatal day; PFC, prefrontal cortex; s.c., subcutaneous injection; SERT, serotonin transporter; Shh, sonic hedgehog; TH, tyrosine hydroxylase; TNF, tumor necrosis factor; TPH, tryptophan hydroxylase; VPA, valproic acid; wks, weeks old.

**Table 2:** Summary of valproic acid (VPA)-induced teratogenicity in catecholaminergic and histaminergic systems.

Monoamines-Related Endophenotypes (Age of Animals during Analysis)	Other Molecular, Cellular, and Physiological Phenotypes (Age of Animals during Analysis)	Behavioral Phenotypes (Age of Animals during Analysis)	Animals	VPA Exposure	References
**Catecholaminergic Systems: Dopamine and Norepinephrine**					
↓ TH immunoreactivity in the striatum (P30)	N/A	↓ Vocalization (P11) ↓ Cognitive flexibility (P29) ↑ Repetitive behavior (P29) ↓ Play behavior (P30)	Wistar rats/male	400 mg/kg, E12.5, i.p.	[131]
↓ TH-positive neurons (5 dpf) ↓ Dopamine β-Hydroxylase (5 dpf and 6 mpf) ↓ DOPAC (5 dpf and 6 mpf) ↓ NE (5 dpf and 6 mpf)	N/A	↓ Locomotion in larvae (5 dpf) Abnormal dark-flash response Abnormal social behaviors (6 mpf)	Zebrafish/male	25 μM, from 10 hpf to 24 hpf, incubated in embryonic medium	[150]
↓ DA in serum (8 wks)	↓ GABA in serum, cerebellum and PFC ↑ Glutamate in serum, cerebellum and PFC ↓ Acetylcholine in serum and PFC Abnormal gut microbiota (8 wks)	↓ Novel object recognition ↓ Social behavior ↓ Locomotion ↑ Depression ↑ Repetitive behavior (8 wks)	Wistar rats/male	500 mg/kg, E12.5, i.p.	[86]
↑ DA in the frontal cortex (P50)	N/A	N/A	SD rats/male	800 mg/kg, E9, oral feeding	[14]
↑ DA level in the dorsal striatum ↓ DA turnover (DOPAC/DA) in the dorsal striatum (1 day after behavioral test)	N/A	Abnormal temporal processing (P60)	CrlFcen:CF1 mice/both gender	600 mg/kg, E12.5, s.c.	[132]
↓ DA in the hippocampus and midbrain ↑ DA in the frontal cortex, cerebellum and pons ↓ NE in the hippocampus and the midbrain ↑ NE in frontal cortex, cerebellum and pons (P22)	↑ Free amino acid in the frontal cortex (P22)	↓ Social behaviors ↓ Spatial memory (P21)	Albino rats/male	800 mg/kg, E12.5, oral feeding	[99]
↑ DA turnover (DOPAC/DA) in the piriform cortex (P60)	↑ c-fos immunoreactivity in the piriform cortex Altered pattern of brain glucose metabolism (P60)	↓ Social behaviors ↑ Repetitive behavior ↑ Anxiety (8 wks)	CrlFcen:CF1 mice/male	600 mg/kg, E12.5, s.c.	[138]
↓ DARPP-32 phosphorylation in response to social stimulus in the nucleus accumbens (P60)	↑ PPARα in the VTA ↑ Vglut and Vglut/Vgat ratio in the caudate-putamen of male mice ↓ PSD95 expression in the caudate-putamen of male mice ↑ NR2B in the caudate-putamen of male mice (P60)	Delayed negative geotaxis ↓ Social behaviors ↑ Repetitive behavior ↑ Depression in male mice ↑ Anxiety (P48–53)	SD rats/both gender	500 mg/kg, E12.5, i.p.	[133]
↑ D1R in the nucleus accumbens and hippocampus ↑ D2R in the nucleus accumbens (P35–40 and P90–95)	↓ Resting potential of medium spiny neurons in the striatum ↓ Excitability ↓ Inwardly rectifying potassium currents density (P30–35)	↓ Social behaviors n.s. amphetamine-induced hyperlocomotion (P35–40)	Wistar rats/male	500 mg/kg, E12.5, i.p.	[134]
↓ *Drd1a* mRNA in the cerebellum ↓ *Drd2* mRNA in the cerebral cortex (P35)	Altered expression in genes encoding cholinergic and adrenergic receptors in the cortex and cerebellum	N/A	Wistar rats/both gender	800 mg/kg, E11, oral feeding	[93]
↓ NE in serum (P28)	↑ Proinflammatory cytokines: IL-17A, TNF-α, IL-6 ↓ GABA in serum Abnormal gut microbiota	Delayed developmental milestones (P2–21) ↓ Locomotion ↓ Social behaviors ↑ Repetitive behavior (P28)	Wistar rats/male	600 mg/kg, E12.5, i.p.	[84]
↑ DAT expression and acetylation of histone H3 bound to *Slc6a3* gene in the PFC ↑ NET expression and acetylation of histone H3 bound to *Slc6a2* gene in the PFC (4 wks)	N/A	Hyperactivity ↑ Repetitive rearing (4 wks)	SD rats/male	400 mg/kg, E12, s.c.	[66]
↓ D1R and D2R in the prefrontal cortex ↓ METH-induced DA release in the prefrontal cortex (8 wks)	↓ c-fos positive neurons in the prefrontal cortex after METH administration (8 wks)	↓ METH-induced hyperlocomotion (8 wks)	ICR mice/male	500 mg/kg, E12.5, i.p.	[135]
Abnormal migration of TH-positive neurons in the pons Abnormal navigation of TH-positive neurons in the boundary of midbrain and hindbrain (E16)	Disorganization of cortical lamination (E16)	N/A	SD rats/both gender	800 mg/kg, E9 or E11, oral feeding	[94]
Abnormal distribution of TH-positive neurons in substantia nigra and VTA ↓ DRD1 and GRIN2A expression in the septum (P2)	N/A	N/A	Chicken (*Gallus gallus*) (N/A)	35 μmoles, E14, dropping VPA solution into the air sac	[136]
↓ Widening TH-positive mesotelecephalic axonal fascicles ↓ TH-positive cells in the VTA ↓ TH-positive terminals in ventrobasal telencephalic region ↑ TH-positive cells in the substantia nigra (P7)	N/A	N/A	C57BL/6/both gender	400 mg/kg, E13.5, s.c.	[137]
**Histamine**					
↓ Histamine receptor H3 ↓ Histidine decarboxylase ↓ Histaminergic neurons (5 dpf and 6 mpf)	N/A	↓ Locomotion in larvae (5 dpf) Abnormal dark-flash response Abnormal social behaviors (6 mpf)	Zebrafish/male	25 μM, from 10 hpf to 24 hpf, incubated in embryonic medium	[150]

All the animals in the studies listed in this table received a single administration of VPA. Abbreviations: ↑, increased; ↓, decreased; D1R, dopamine D1 receptor; D2R, dopamine D2 receptor; DAT, dopamine transporter; DOPAC, 3,4-dihydroxyphenylacetic acid; dpf, days post fertilization; E, embryonic day; GABA, γ-Aminobutyric acid; hpf, hours post-fertilization; IL, interleukin; i.p., intraperitoneal injection; METH, methamphetamine; mpf, months post-fertilization; m/o, months old; n.s., no significant difference; N/A, not available; NR2B, N-methyl D-aspartate receptor subtype 2B; P, postnatal day; PFC, prefrontal cortex; PPARα, peroxisome proliferator-activated receptor alpha; PSD95, postsynaptic density protein 95; s.c., subcutaneous injection; TH, tyrosine hydroxylase; TNF, tumor necrosis factor; VPA, valproic acid; VTA, ventral tegmental area; wks, weeks old.
